# Carotid dissection and central serous chorioretinopathy related to sarcoidosis-antiphospholipid syndrome: a case report

**DOI:** 10.22336/rjo.2022.38

**Published:** 2022

**Authors:** Francisco Javier Valentín-Bravo, Luis García-Onrubia, Miguel Martín-Asenjo, Jorge Galván-Fernández, Salvador Pastor-Idoate

**Affiliations:** * Department of Ophthalmology, University Clinical Hospital of Valladolid, Valladolid, Spain; ** Department of Ophthalmology, St. Thomas’ Hospital, London SE1 7EH, UK; *** Department of Internal Medicine, University Clinical Hospital of Valladolid, Valladolid, Spain; **** Department of Radiology, University Clinical Hospital of Valladolid, Valladolid, Spain; ***** Institute of Applied Ophthalmobiology (IOBA), University of Valladolid, Valladolid, Spain; Cooperative Network for Research in Ophthalmology (Oftared), Carlos III National Institute of Health, ISCIII, Madrid, Spain

**Keywords:** central serous chorioretinopathy, carotid dissection, sarcoidosis, antiphospholipid syndrome

## Abstract

Sarcoidosis is a chronic multisystemic disease, which can be rarely associated with autoimmune disorders, such as antiphospholipid syndrome (APS). Although amaurosis fugax is an uncommon complication, its presentation can unmask a carotid artery dissection (CAD) in these diseases. In addition, central serous chorioretinopathy (CSC) has been related to vascular disorders too.

We presented a case of a Caucasian middle-aged man, who developed CAD symptoms, such as amaurosis fugax in the right eye (RE) and headache. His medical history included arterial hypertension, hypothyroidism, and Lofgren’s syndrome. On examination, retinal pigment epithelium (RPE) atrophy and subretinal fluid (SRF) in the macular area of the RE were observed. These findings were confirmed by optical coherence tomography (OCT), which also revealed an increase in choroidal thickness. However, these differed significantly from the contralateral eye. These clinical symptoms and imaging findings suggested a CSC in the RE, but not all clinical processes were justified. Subsequently, a CT angiography was performed and confirmed a significant occlusion in the right internal carotid artery and progressive sharpening of the lumen with an intimal flap due to a carotid dissection. In addition, the laboratory results were compatible with antiphospholipid syndrome (APS). To the authors’ knowledge, the patient returned to the ED due to an anterior uveitis and he is currently asymptomatic with Cemidon and Adalimumab treatment.

We described for the first time a case of carotid dissection and central serous chorioretinopathy in the context of two autoimmune-based pathologies, such as sarcoidosis and antiphospholipid syndrome.

**Abbreviations:** APS = Antiphospholipid syndrome, BCVA = Best-corrected visual acuity, CAD = Carotid artery dissection, CNV = Choroidal neovascular membrane, CSC = Central serous chorioretinopathy, CT = Computed tomography, ED = Emergency Department, ICAD = Internal carotid artery dissection, LE = Left eye, OCT = Optical coherence tomography, RAPD = Relative afferent pupillary defect, RPE = Retinal pigment epithelium, RE = Right eye, SRF = Subretinal fluid

## Introduction

Sarcoidosis is a chronic multisystemic disease with unknown etiology that can potentially affect any organ, characterized by non-caseating granulomatous inflammation [**1**,**2**]. It can be associated with autoimmune disorders such as antiphospholipid syndrome (APS), however being a rare association [**2**].

APS or Hughes syndrome is an acquired autoimmune disease defined by the persistence of antiphospholipid antibodies and characterized by recurrent thrombotic events [**3**]. Although amaurosis fugax is a rare complication occurring in 7% of patients with APS, its presentation can unmask serious events such as thrombus or carotid dissection, which is not an uncommon cause of stroke in young adults [**3**].

On the other hand, central serous chorioretinopathy (CSC) is an idiopathic disease characterized by neurosensory detachments with subretinal fluid accumulation. The exact etiology remains poorly understood, and several risk factors have been studied, such as vascular disturbances [**4**].

Each internal carotid artery carries blood flow to its ipsilateral eye, so unilateral ophthalmologic disturbances/ disorders could be the initial symptom of internal carotid artery dissection (ICAD).

The article describes, for the first time, a case of carotid dissection and central serous chorioretinopathy in the context of two autoimmune-based pathologies, such as sarcoidosis and antiphospholipid syndrome.

## Clinical Case

A 54-year-old Caucasian male presented to the Emergency Department (ED) with complaints of unilateral oppressive headache in the right frontal region, relieved after taking analgesics. He reported episodes of acute transient monocular visual loss (also mentioned as amaurosis fugax) twice, with spontaneous and complete recovery after 1-2 minutes on the right eye.

Among his family antecedents, his mother suffered from rhupus. The personal medical history included arterial hypertension, hypothyroidism, and he also suffered from Löfgren’s syndrome seven years earlier. 

**Fig. 1:**
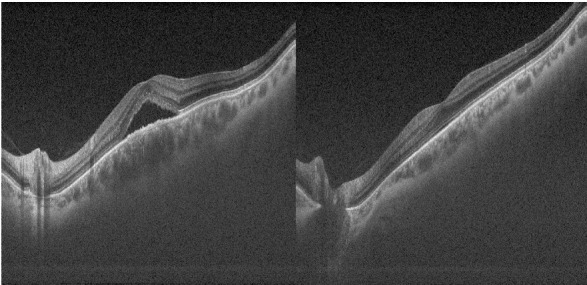
Optical coherence tomography (OCT) showing retinal pigment epithelium (RPE) atrophy and the presence of subretinal fluid (SRF) in the macular area of the right eye. In addition, a pachychoroid was observed and its thickness differed significantly from the contralateral

In the ED, neurological and physical examinations exposed no noteworthy findings. Ophthalmological tests showed normoreactive isochoric pupils, without relative afferent pupillary defect (RAPD). Best-corrected visual acuity (BCVA) was 20/ 25 in the right eye (RE) and 20/ 20 in the left eye (LE), and slit-lamp examination revealed normal anterior segment findings in both eyes. However, dilated fundus examination and the optical coherence tomography (OCT) showed retinal pigment epithelium (RPE) atrophy and the presence of subretinal fluid (SRF) in the macular area (**Fig. 1**). In addition, a pachychoroid was observed, and its thickness differed significantly from the contralateral (**Fig. 1**). These clinical symptoms and imaging findings suggested a CSC in the RE, but not all clinical processes were justified.

**Fig. 2:**
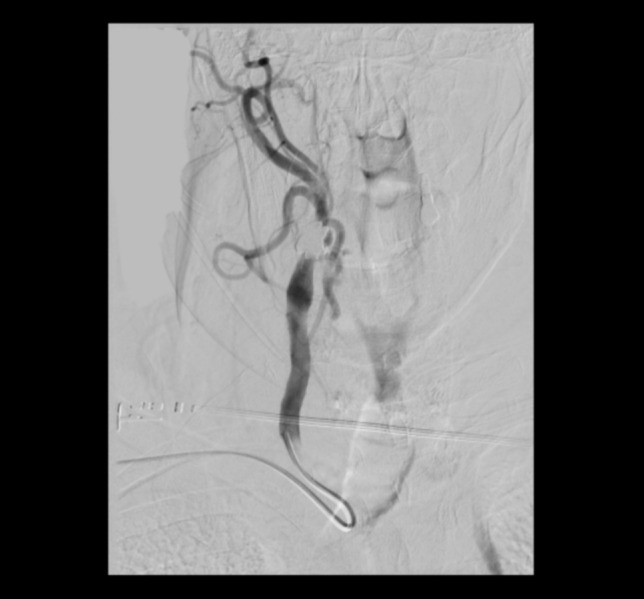
The CT angiography confirmed a significant occlusion in the right internal carotid artery and progressive sharpening of the lumen with an intimal flap due to a carotid dissection

Therefore, a blood test with coagulation, a brain computed tomography (CT) scan, and a carotid ultrasound were requested to clarify the episodes of amaurosis fugax described by the patient. The CT angiography confirmed a significant occlusion in the right internal carotid artery and progressive sharpening of the lumen with an intimal flap due to a carotid dissection (as it can be observed in **Fig. 2**). Duplex ultrasound showed findings of the supra-aortic trunks that were compatible with an occlusion of the distal right carotid axis at the ophthalmic level. Echocardiogram and electrocardiogram were negative for embolic source, valvular abnormality, foramen ovale, or cardiac arrhythmia.

Due to these findings, the patient was admitted, and dual antiplatelet therapy consisting of aspirin 300 mg and clopidogrel 75 mg once daily were started for stroke prevention. 

A laboratory investigation was performed and showed a positive result for lupus anticoagulant, which was positive again at three months. These results were compatible with antiphospholipid syndrome (APS).

To the authors’ knowledge, one year later, the patient presented to the ED again with ocular pain, photophobia, and conjunctival hyperaemia. The ophthalmic findings at the time of that examination were a clear cornea and 1.5 + anterior chamber cells in the RE. He was diagnosed with anterior uveitis, possibly secondary to systemic pathologies. In addition, he presented pain and inflammation in the hands and a panniculitis lesion in the left leg, so it was thought that sarcoidosis had been activated. 

Therefore, treatment with Cemidon 300 mg every 24 h and Adalimumab 40 mg subcutaneous every 15 days was started. He was not treated with corticosteroids due to the presence of previous CSC. Currently, he is asymptomatic and his OCT images show no subretinal or intraretinal fluid.

## Discussion

This case described a middle-aged man, who developed symptoms of carotid artery dissection (CAD), such as amaurosis fugax and headache. CAD is known to be an important cause of ischemic stroke in young and middle-aged patients [**5**]. Although the pathogenesis remains unknown, numerous risk factors have been postulated, and this condition can be related to underlying autoimmune disorders, such as sarcoidosis and APS [**1**,**6**-**12**].

Most frequent ocular manifestations in APS are vascular occlusion and retinal vasculitis, so, these patients could present with amaurosis fugax, whose prevalence is 5.4% [**6**-**12**]. It has been suggested that interaction between antiphospholipid antibodies and endothelial cells may contribute to the dissection and the local vasculitis [**13**]. Currently, Iseki et al. (2020) [**14**] collected eight published cases of CAD secondary to APS, similar to our case. 

In addition, choroidal neovascular membranes (CNV) and CSC have been associated with APS too, as we presented in our case [**6**]. These CSC can be caused by choroidal circulation disturbance that leads to subretinal leakage, being usually related to catastrophic APS [**6**,**15**]. The hypothesis of choroidal capillary thrombosis leading to subretinal leakage was raised by the authors. 

The sarcoidosis implication, proposed by Mikami et al. [**16**], can also be added to the vascular damage (microangiopathy). Moreover, it is also true that currently, Ari et al. reported a rare case of proximal carotid occlusion in the case of sarcoidosis [**17**]. 

Although sarcoidosis is an inflammatory disorder that usually presents with hilar lymphadenopathy and lung infiltration, multiple organs such as the eyes may be involved in more than half of the patients [**18**,**19**], causing pathologies that can lead to blindness [**20**]. Neurological involvement occurs only in 5% of cases, and sarcoidosis uveitis is a more prevalent manifestation that usually presents as chronic and bilateral condition [**21**]. Despite this issue, our patient only suffered from one acute unilateral anterior chamber inflammation and sarcoidosis may contributed to the previous clinical picture. 

In addition, although our patient was a male, females are more likely to develop autoimmune diseases and ocular involvement [**20**]. Consequently, Pathak et al. [**2**] included four cases of sarcoidosis with APS, and all of them were females. 

Another problem to be tackled is that we cannot overlook that corticosteroids are one of the most used treatments in rheumatologic conditions and can cause CSC as an adverse event.

CT angiography has been the gold standard for diagnosing carotid dissection and is mandatory (or must be performed) in every case of amaurosis fugax. Hence, an intimal flap may be revealed, as described in this case.

Furthermore, OCT is indispensable for the diagnosis and monitoring of microvascular changes to rule out an ocular pathology, such as CSC.

Moreover, these patients should be studied in detail in order to look for an underlying systemic cause, with a complete examination and complementary test, such as an echocardiogram and lab test.

We believe that the clinical suspicion and a multidisciplinary approach is the key to reach a definitive diagnosis in these complex cases. In addition, management should not be delayed, and is usually non-surgical, with antiplatelets or anticoagulation drugs. 

Laser photocoagulation and photodynamic therapy with verteporfin can be used as a treatment in CSC.

To our knowledge, there is no published case in which sarcoidosis is associated with APS and carotid dissection in a male patient. In our opinion, CSC is under-diagnosed in the APS patient, according to its mild level compared to other APS symptoms, such as Billoir et al. underlined.

## Conclusion

Patients with signs of amaurosis fugax and increased choroidal thickness should be studied in detail in order to rule out vascular lesions and associated autoimmune diseases. A multidisciplinary approach is necessary for the early detection and management of these patients.

**Conflict of Interest statement**

Authors state no conflict of interest.

**Informed Consent and Human and Animal Rights statement**

Informed consent has been obtained from the individual included in this study, who agreed to the publication of this case.

**Authorization for the use of human subjects**

Ethical approval: The research related to human use complies with all the relevant national regulations, institutional policies, is in accordance with the tenets of the Helsinki Declaration, and has been approved by the review board of University Clinical Hospital of Valladolid, Valladolid, Spain.

**Acknowledgements**

None.

**Sources of Funding**

None.

**Disclosures**

None.
